# When Saying “Yes” Hurts: The Relationship Between Compliance and Psychological Distress Across Cultural Values in China

**DOI:** 10.3390/bs16071187

**Published:** 2026-07-14

**Authors:** Dan Dong, Ziyi Yan, Yi Feng, Aiyi Liu, Zhihong Qiao

**Affiliations:** 1 School of Psychology, Fujian Normal University, Fuzhou 350117, China; dongdan92@fjnu.edu.cn; 2 National Demonstration Center for Experimental Psychology Education, Faculty of Psychology, Beijing Normal University, Beijing 100089, China; 202521061093@mail.bnu.edu.cn; 3 Mental Health Center, Central University of Finance and Economics, Beijing 100089, China; 4 School of Sociology and Psychology, Central University of Finance and Economics, Beijing 100089, China; 5 State Key Laboratory of Cognitive Science and Mental Health, Institute of Psychology, Chinese Academy of Sciences, Beijing 100089, China; 202131061014@mail.bnu.edu.cn

**Keywords:** compliance, psychological distress, positive affect, negative affect, individualism-collectivism

## Abstract

Background: Young adult mental health is a critical global concern. This study aims to investigate the relationship between compliance and psychological distress in young adults, paying particular attention to the mediating role of affect and the moderating role of cultural values in China. Methods: A large-scale cross-sectional study was conducted, with 11,038 (Mage = 20.58 ± 4.11) young adults recruited. A series of self-reported questionnaires was used, including measures of psychological distress, cultural factors, and compliance. Latent structural equation modelling was applied to investigate how the compliance trait links to psychological distress. Results: Findings revealed a positive association between compliance and psychological distress. The analysis further demonstrated that both positive and negative affect served as significant mediators in this association. Additionally, the association was moderated by cultural factors, particularly individualism. Individualism significantly buffered both the direct effect of compliance on psychological distress and the mediating effect of negative affect. Conclusions: These results contribute to a deeper understanding of the social and psychological determinants of youth mental health, a key priority for public health practice.

## 1. Introduction

In everyday social interactions, complying with others’ requests can often be as simple as saying ‘yes,’ whether to a friend’s plea for help, a colleague’s request for extra work, or a family member’s expectation. While such acquiescence may appear harmless on the surface, a dispositional tendency to prioritize others’ needs over one’s own can carry psychological costs.

Compliance, typically defined as the tendency to comply with others’ requests, demands, or social pressure, has long been a pivotal concept in psychological research (Bond & Smith, 1996; Nail, 1986). It has been extensively examined across several areas of psychology, including social, developmental, and clinical psychology (Bond & Smith, 1996; Nail, 1986). Previous investigations into compliance within these realms have predominantly focused on situational compliance—immediate behavioral responses to requests from others, often referred to as compliance behavior (Kosslyn & Rosenberg, 2005). However, some scholars have proposed that individuals differ in their responses to social pressures and others’ demands, and that these tendencies remain relatively stable over time (Gudjonsson et al., 2002, 2008). Those characterized by high compliance tendencies are more likely to comply with others’ requests or demands in their daily interactions, suggesting that compliance can be conceptualized as a stable dispositional trait (Gudjonsson et al., 2002). Trait compliance thus denotes an individual’s propensity to comply with others’ requests for personal gain (Gudjonsson, 1992). This study aims to examine compliance through a trait-based perspective, thereby enriching its theoretical framework and advancing our understanding of this psychological phenomenon.

### 1.1. Compliance and Psychological Distress

Among the multitude of factors influencing psychological distress, personality traits have consistently been identified as important contributors (Kotov et al., 2010; Malouff et al., 2005; Ormel et al., 2013; Ozer & Benet-Martinez, 2006). As a stable dispositional trait, compliance may serve as a vulnerability factor for psychological distress. Drawing on self-determination theory (Ryan & Deci, 2017), we propose that the association between compliance and psychological distress operates through the frustration of the basic need for autonomy. When individuals habitually prioritize others’ demands over their own authentic preferences, they are more likely to experience autonomy frustration, which undermines psychological well-being (Ryan & Deci, 2024) and contributes to psychological distress. From the perspective of self-esteem, this autonomy frustration may further foster contingent self-esteem, which is a fragile form of self-worth that depends on external approval (Moller et al., 2006; Ryan & Deci, 2000). Individuals high in compliance tend to seek social validation at the expense of their own preferences (Gudjonsson et al., 2002), perpetuating a cycle in which contingent self-esteem heightens vulnerability to psychological distress (Cambron et al., 2010).

### 1.2. Compliance, Affect and Psychological Distress

The close relationship between personality traits and affect has been substantiated by extensive research (Kang et al., 2023; Wu, 2023). Notably, a robust association exists between compliance and affect (Dolinski, 2001). Some scholars have emphasized the importance of considering the affective mechanisms and processes in compliance research (Gudjonsson et al., 2008).

Affective experiences refer to the subjective feelings and emotional responses that individuals encounter in their daily lives (Gross, 2015; Scherer, 2005). Positive affect and negative affect represent the two main dimensions of affective experience (Watson et al., 1988). Positive affect encompasses pleasant and energizing emotional states, such as energy, excitement, and concentration (Watson et al., 1988). Negative affect, by contrast, reflects aversive emotional states, including anger, sadness, tension, fear, and other unpleasant emotional experiences (Watson et al., 1988). Positive affect and negative affect are not opposite ends of a single continuum but rather two distinct and relatively independent dimensions (Crawford & Henry, 2004; Watson & Tellegen, 1985). It means that experiencing high levels of positive affect does not necessarily mean experiencing low levels of negative affect, and vice versa. Individuals can simultaneously experience both positive and negative affect to varying degrees, resulting in complex affective states.

Similarly, according to self-determination theory (Ryan & Deci, 2017), when individuals habitually cater to others’ demands and suppress their true preferences, their behavior is driven by external pressure rather than personal choice, resulting in autonomy frustration. This frustration may generate emotional conflict and negative affect, as individuals perceive a discrepancy between their authentic selves and their compliant behavior (Leary et al., 1995). Indeed, compliance-related pressures have been shown to evoke a range of negative emotions, including fear, anger, guilt, and disappointment, which may persist throughout the compliance process (Gudjonsson et al., 2002). Moreover, when compliance is experienced as externally imposed rather than autonomously chosen, it may undermine intrinsic motivation and diminish positive affect, while amplifying negative affect (Brockner et al., 1992; Ryan & Deci, 2017).

Sustained positive affect is linked to various mental health benefits, including improved physical health, longevity, work satisfaction, and social relationships (Lyubomirsky et al., 2005). Conversely, negative affect is a core feature of many psychological disorders, such as depression, anxiety, and post-traumatic stress disorder (Kessler et al., 2005). When negative affect persists or becomes intense, it can also pose a risk to mental health. Therefore, it is reasonable to posit that affect is a mechanism linking compliance to psychological distress.

### 1.3. Culture Matters in the Association Between Compliance and Psychological Distress

The detrimental effects of compliance on psychological distress have primarily been studied in individualistic cultural contexts (Gudjonsson & Main, 2008; Levy & Gudjonsson, 2006). Individualism is characterized by a greater emphasis on individual rights, freedoms, needs, and identity (Branzei et al., 2007; Menon et al., 1999; Morris & Peng, 1994; Triandis, 2011). In contrast, collectivism pertains to cultural contexts where greater emphasis is placed on the rights, needs, and identity of the group (Triandis, 2018). Given the emphasis on group cohesion and harmony in collectivist cultures (Triandis, 2018), compliance may be perceived and function differently, potentially showing distinct associations with psychological distress. Therefore, exploring the role of compliance in psychological distress within collectivist cultural contexts is essential for a comprehensive understanding of this phenomenon.

In cultural contexts that prioritize individual goals and rights, values such as independence, personal autonomy, and self-fulfillment are strongly emphasized (Feng et al., 2023; Zhang, 2024). Social relationships are typically based on individual preferences and needs (Zhang, 2024). However, compliance often entails sacrificing personal interests, which may conflict with the core values of such contexts (Zhang, 2024). Consequently, among individuals with strong individualistic tendencies, higher compliance may be associated with reduced perceived control, heightened negative affect, and diminished positive affect, which are in turn associated with psychological distress. Conversely, in cultural contexts that emphasize group harmony and collective welfare, individual behaviors and choices are often shaped by group interests and expectations (Hofstede, 1984). Thus, in such contexts, compliance may be more closely aligned with fulfilling interpersonal needs and social obligations, thereby promoting greater positive affect and lower negative affect. This pattern may in turn be associated with lower levels of psychological distress among individuals with high collectivist tendencies. Therefore, the relationship between compliance and psychological distress, as well as the underlying affective mechanisms, may vary according to individual-level cultural orientations.

### 1.4. Aim of This Study

This study aims to examine the relationship between compliance and psychological distress, explore the underlying affective mechanism, and investigate the moderating role of cultural values in this relationship. Accordingly, we addressed the following research questions: (a) whether trait compliance is positively associated with psychological distress in Chinese young adults, (b) whether positive and negative affect mediate this relationship, and (c) whether individualism and collectivism moderate the direct and/or indirect effects of compliance on psychological distress. We hypothesized that compliance is positively associated with psychological distress; higher compliance is associated with psychological distress through more negative affect and less positive affect; and compared to individuals with lower individualism and higher collectivism, those with higher individualism and lower collectivism will exhibit a stronger direct relationship between compliance and psychological distress, as well as an intensified mediating effect of affect.

Importantly, these hypotheses are grounded in the assumption that cultural values operate at the individual level, reflecting within-culture variation rather than cross-cultural differences between societies.

## 2. Method

### 2.1. Participants

This study employed a cross-sectional design and was conducted in September 2023. The minimum sample size was determined based on the 10:1 rule of thumb for structural equation modeling (Grace, 2006), requiring a minimum of 1400 participants for approximately 140 parameters. A total of 12,535 students were recruited from three universities in Beijing, Xiamen, and Ningxia Province, China, using cluster sampling. After applying the predefined exclusion criteria, 1497 participants were excluded due to missing data or invalid responses, yielding a final sample of 11,038 students (Mean age = 20.58 ± 4.11 years), with a completion rate of 88.1%. Of the participants, 66.6% were female, 87.8% were of Han ethnicity, and 60.1% were university students (see Table 1 for details).

The exclusion criteria included: (a) discontinuation of participation during the survey; (b) an average response time of less than one second per question; and (c) incorrect responses to more than three attention test questions. Data were handled using listwise deletion, as the proportion of missing responses was minimal (<2% per variable).

Participants completed the questionnaire online, with an average completion time of approximately 20–25 min. Before participation, all respondents provided informed consent, which included a detailed explanation of the study’s objectives and their right to withdraw at any time. Ethical approval for the study was granted by the Research Ethics Review Committee of Central University of Finance and Economics, China.

### 2.2. Measures

#### 2.2.1. Social-Demographic Variables

Participants’ sociodemographic characteristics were assessed using a set of self-developed items, covering age, sex, ethnicity, education level, type of residence, only-child status, psychiatric history, current medication use, and family economic status (see Supplementary Materials for details). In addition, subjective social class was measured using a widely adopted 10-rung ladder measure, where participants indicated their perceived social standing, with 0 representing the lowest social position and 10 the highest (Sainz et al., 2021).

#### 2.2.2. Compliance

Compliance was assessed using the Chinese version of the Compliance Scale (Hang et al., 2024), adapted from the Gudjonsson Compliance Scale (GCS) (Gudjonsson, 1989). The scale consists of 18 items. Participants rated their compliance tendencies by responding to statements such as “I would describe myself as a very obedient or compliant person” and “When I am under pressure, I easily compromise with others.” To improve measurement sensitivity, the Chinese version adopted a 5-point Likert scale ranging from 1 (“completely disagree”) to 5 (“completely agree”). Three items were reverse-scored. The total score across all 18 items was calculated, with higher scores indicating greater trait compliance. The scale demonstrated strong internal consistency in this study (Cronbach’s *α* = 0.89).

#### 2.2.3. Psychological Distress

Psychological distress was assessed with the Symptom Checklist-90 (SCL-90) (Derogatis et al., 1973). This scale comprises 10 dimensions: somatization, obsessive–compulsive symptoms, interpersonal sensitivity, depression, anxiety, hostility, phobic anxiety, paranoid ideation, psychoticism, and additional items (primarily appetite and sleep). Participants were asked to report the extent to which they had experienced symptoms of psychological distress in the past week, such as “headaches” or “unnecessary thoughts or words swirling in their minds”. This scale employs a 5-point Likert scoring system (1 = “none”; 5 = “extremely”). Scores for each dimension were summed separately, with higher scores indicating more severe symptoms of the respective psychological distress. In this study, the Cronbach’s *α* coefficients of the scale and its subscales ranged from 0.76 to 0.98.

#### 2.2.4. Positive and Negative Affect

Positive and negative affect were assessed using the Positive and Negative Affect Schedule (PANAS) (Watson et al., 1988). Participants rated the extent to which they had experienced each specific affect over the past two weeks using a 5-point Likert scale, ranging from 1 (“none at all”) to 5 (“a lot”). The scale comprises 20 items, with 10 items measuring positive affect (e.g., “excited”) and 10 items measuring negative affect (e.g., “depressed”). Total scores for the positive and negative affect subscales were calculated separately, with higher scores indicating higher levels of the corresponding affect. In this study, both the positive affect (Cronbach’s *α* = 0.90) and negative affect (Cronbach’s *α* = 0.90) subscales demonstrated high internal consistency reliability.

#### 2.2.5. Individualism–Collectivism

Individualism and Collectivism was assessed using the Individualism and Collectivism Scale (Singelis et al., 1995). The scale consists of 32 items organized into two dimensions: individualism and collectivism, each with 16 items. Participants rated their agreement with statements on a 5-point Likert scale (1 = “completely disagree”; 5 = “completely agree”). Example items include “I am a unique individual”, and “I often sacrifice my own interests for the collective benefit”. Higher scores on each dimension indicate a stronger individualistic or collectivistic orientation. In this study, the individualism subscale (Cronbach’s *α* = 0.74) and collectivism subscale (Cronbach’s *α* = 0.79) both demonstrated high internal consistency.

### 2.3. Statistical Analysis

Data were analyzed using IBM SPSS 23.0 (IBM Corp., Armonk, NY, USA) and Mplus 8.3 (IBM Corp., Armonk, NY, USA), and figures were generated using RStudio 2023.06.1 (Posit Software, PBC, Boston, MA, USA). First, a Common Method Bias (CMB) test was conducted using Harman’s single-factor test by entering all measurement items into an unrotated exploratory factor analysis. Second, the descriptive statistics were calculated, and Pearson correlations among the main variables were examined. Third, a direct model was established to examine the direct effect of compliance on psychological distress, with latent compliance as the predictor, latent psychological distress as the outcome, and sociodemographic variables as covariates. Fourth, based on the direct model, positive and negative affect were added as mediating variables to construct a latent mediation model. Fifth, individualism and collectivism were entered separately as moderating variables in the latent mediation model to examine their moderating effects on the relationship between compliance and psychological distress, as well as the mediating effect of positive and negative affect. All categorical variables were coded as dummy variables in all data analyses.

The latent moderation effect test involved two steps. First, Model 0 (without interaction terms of compliance and individualism/collectivism) was estimated, and model fit was evaluated using *χ*^2^/*df*, RMSEA, CFI, TLI, and SRMR (Hu & Bentler, 1998). Model fit was considered acceptable when RMSEA < 0.08, SRMR < 0.08, CFI > 0.90, and TLI > 0.90 (Wen et al., 2004). Second, based on Model 0, a latent moderation model (Model 1) was constructed by adding a latent interaction term between compliance and individualism/collectivism. A log-likelihood ratio test was used to determine whether the latent moderation model was superior to Model 0. A significant test result indicates that the latent moderation model provides a better fit (King, 1989).

It is important to note that, given the cross-sectional design, all reported direct and mediation effects are correlational in nature and should be interpreted as associations rather than causal relationships.

## 3. Results

### 3.1. Common Method Bias and Correlations Between Main Variables

**Figure 1:**
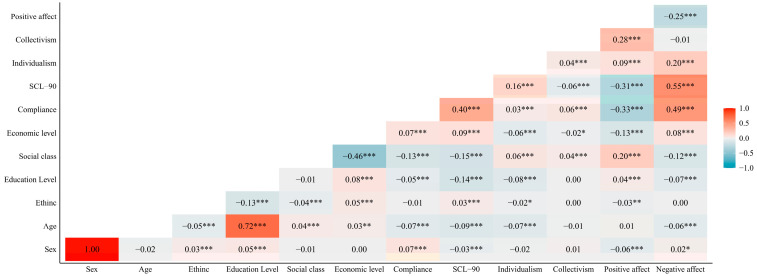
Correlations among the main variables (*N* = 11,038). Note. * *p* < 0.05, ** *p* < 0.01, *** *p* < 0.001.

The results of the common method bias test showed that the first unrotated factor accounted for 24.08% of the total variance. This value is below the 40% threshold, indicating that common method bias was not a serious concern (Podsakoff et al., 2003). The correlation analysis results (see Figure 1) revealed that SCL-90 scores were moderately correlated with compliance (*r* = 0.40, *p* < 0.001), positive affect (*r* = −0.31, *p* < 0.001), and negative affect (*r* = 0.55, *p* < 0.001). Additionally, compliance was significantly correlated with both positive affect (*r* = −0.33, *p* < 0.001) and negative affect (*r* = 0.49, *p* < 0.001). Given the large sample size, we evaluated the practical significance of these associations by examining effect sizes (Cohen, 2013). All core correlations fell within the small-to-medium to medium range (|*r*| = 0.31–0.55), accounting for approximately 10% to 30% of the shared variance, suggesting that these associations were practically meaningful. The correlations between SCL-90 and the two cultural dimensions (individualism and collectivism) were relatively weak (*rs* = 0.16 and −0.06, respectively), which is consistent with their role as moderators rather than direct predictors in our theoretical model.

### 3.2. Relationship Between Compliance and Psychological Distress

**Figure 2:**
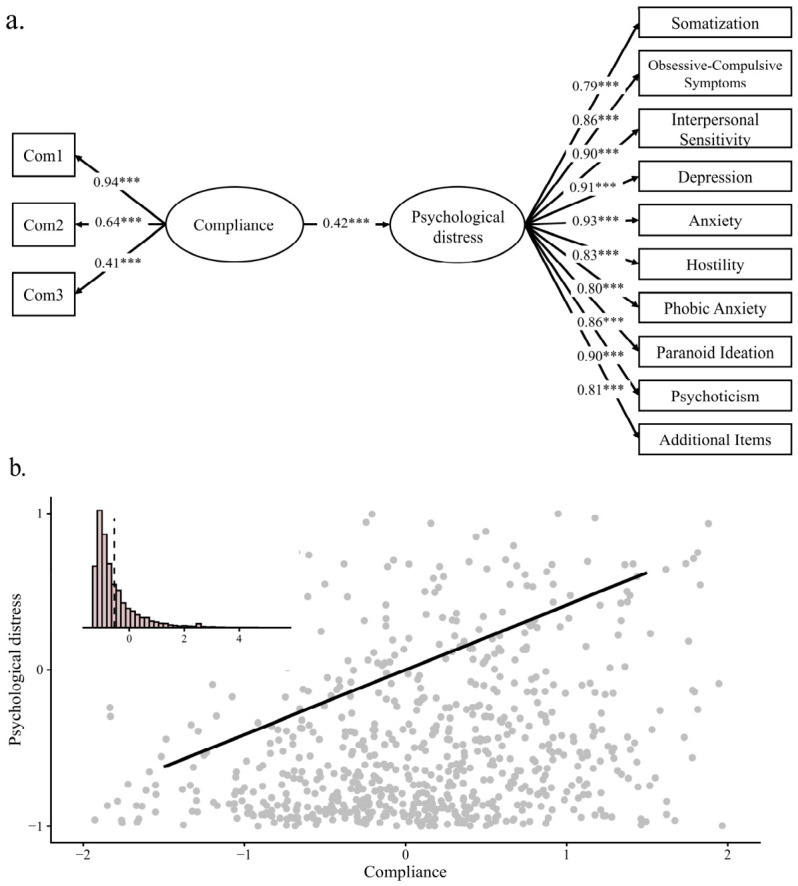
(**a**) Latent direct structural equations model (*N* = 11,038). Note. Com1–Com3 are items from the compliance scale. *** *p* < 0.001. (**b**) The predictive effect of compliance on psychological distress. Note. The solid line represents the standardized regression coefficient from compliance to psychological distress in the SEM model. The scattered points are randomly selected latent variable scores for both variables from 10% of the samples. The subplot in the upper left corner shows the distribution of the latent variable scores of psychological distress. The dashed vertical line indicates the mean of the latent variable scores for psychological distress.

A latent direct structural equations model was constructed with sociodemographic variables as covariates, compliance as the latent independent variable, and psychological distress as the latent dependent variable (see Figure 2a). The model demonstrated good fit: χ^2^/*df* = 66.122, CFI = 0.933, TLI = 0.923, RMSEA = 0.077, 90% CI = [0.075, 0.078], SRMR = 0.042. Compliance was significantly and positively associated with psychological distress (*β* = 0.42, 95% CI = [0.39, 0.43], *p* < 0.001) (see Figure 2b).

### 3.3. Mediating Effects of Positive and Negative Affect

**Figure 3:**
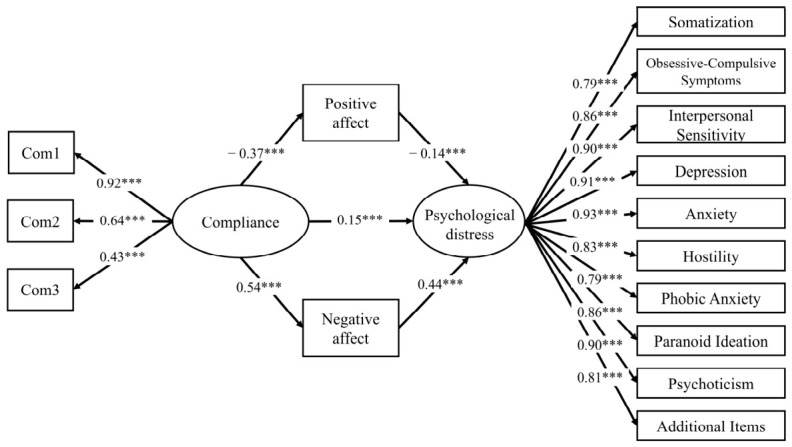
Latent mediation structural equations model (*N* = 11,038). Note. Com1–Com3 are compliance scale items. *** *p* < 0.001.

Based on the latent direct model, a latent mediation structural equations model was constructed by adding positive and negative affect as mediating variables (see Figure 3). The model demonstrated good fit: *χ*^2^/*df* = 64.280, CFI = 0.924, TLI = 0.913, RMSEA = 0.076, 90% CI [0.075, 0.077], SRMR = 0.050. Compliance was negatively associated with positive affect (*β* = −0.37, 95% CI = [−0.39, −0.34], *p* < 0.001) and positively associated with negative affect (*β* = 0.54, 95% CI = [0.52, 0.55], *p* < 0.001). Furthermore, positive affect was negatively associated with psychological distress (*β* = −0.14, 95% CI = [−0.16, −0.12], *p* < 0.001), whereas negative affect was positively associated with psychological distress (*β* = 0.44, 95% CI = [0.42, 0.46], *p* < 0.001). The indirect effects of compliance on psychological distress via positive affect (*β* = 0.05, 95% CI = [0.04, 0.06], *p* < 0.001) and negative affect (*β* = 0.23, 95% CI = [0.22, 0.25], *p* < 0.001) were both significant. Positive affect and negative affect accounted for 0.23 and 0.43 of the total effect, respectively.

### 3.4. Moderating Effects of Individualism–Collectivism on Direct Effect

Based on the latent direct structural equation model, we established a latent moderation structural equation model by adding individualism, collectivism, and their latent interaction terms with compliance as predictors to test the moderating effects of individualism and collectivism on the direct relationship. The results showed that Model 0 without interaction terms fit well (Individualism: *χ*^2^/*df* = 64.759, CFI = 0.929, TLI = 0.919, RMSEA = 0.076, 90% CI [0.075, 0.077], SRMR = 0.042; Collectivism: χ^2^/*df* = 65.967, CFI = 0.928, TLI = 0.918, RMSEA = 0.077, 90% CI [0.075, 0.078], SRMR = 0.043).

**Figure 4:**
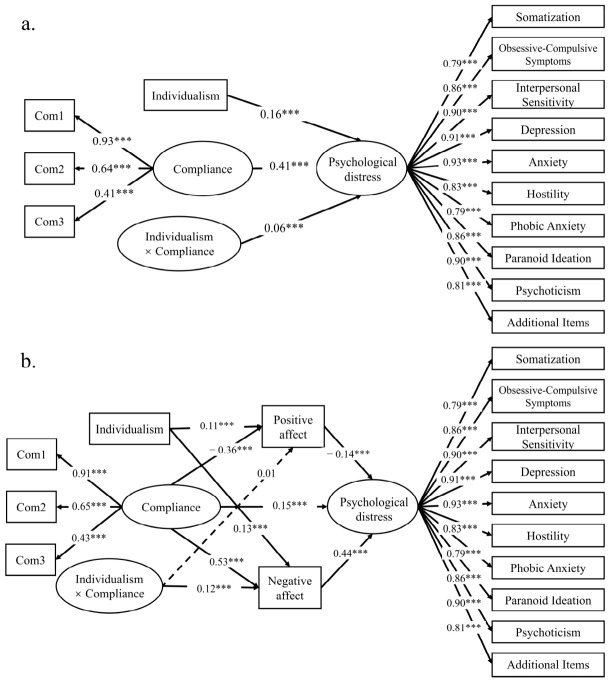
(**a**) Latent moderated structural equations model. Note. Com1–Com3 are compliance scale items. *** *p* < 0.001. (**b**) Latent moderated-mediation structural equations model. Note. Com1–Com3 are compliance scale items. *** *p* < 0.001.

The model including the interaction between compliance and individualism (see Figure 4a) yielded a log-likelihood ratio of *D* = 58.74, exceeding the critical value of 3.84 (*df* = 1), indicating that the individualism-moderated model provided a significantly better fit than Model 0. Correspondingly, the moderating effect of individualism on the relationship between compliance and psychological distress was significant (*β* = 0.06, 95% CI = [0.05, 0.08], *p* < 0.001). In contrast, the model with the interaction between compliance and collectivism produced a log-likelihood ratio of *D* = 1.00, which does not exceed the 3.84 threshold (*df* = 1), indicating that the collectivism-moderated model does not offer a superior fit over Model 0. Consistently, the moderating effect of collectivism was not significant (*β* = 0.01, 95% CI = [−0.01, 0.03], *p* = 0.313).

**Figure 5:**
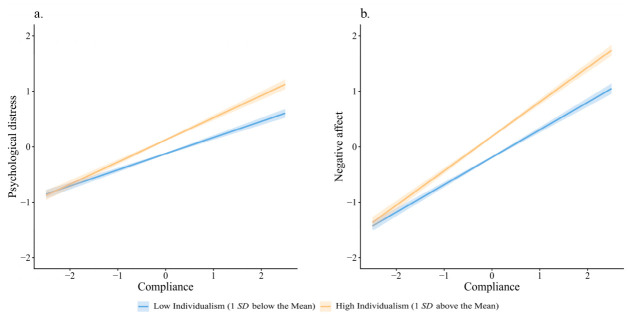
(**a**) Moderating effect of individualism on psychological distress. (**b**) Moderating effect of individualism on negative affect.

Simple slope analysis revealed that, compared with low individualism (−1 *SD*) (*b* = 0.29, 95% CI = [0.27, 0.31], *p* < 0.001), the positive association between compliance and psychological distress is stronger for high individualism (+ 1 *SD*) (*b* = 0.40, 95% CI = [0.37, 0.43], *p* < 0.001) (see Figure 5a).

### 3.5. Moderating Effects of Collectivism–Individualism on Mediating Effects

Based on the latent mediation model, we constructed a latent moderated mediation model by adding collectivism and individualism as moderators to test their moderating effects on the mediating effects of positive and negative affect. The results indicated that Model 0, without interaction terms between compliance and the two cultural dimensions, demonstrated acceptable fit (Individualism: χ^2^/*df* = 63.513, CFI = 0.919, TLI = 0.908, RMSEA = 0.075, 90% CI [0.074, 0.076], SRMR = 0.052; Collectivism: χ^2^/*df* = 53.311, CFI = 0.913, TLI = 0.891, RMSEA = 0.069, 90% CI [0.067, 0.071], SRMR = 0.063).

The model including the interaction between compliance and individualism (see Figure 4b) yielded a significant log-likelihood ratio (*D* = 49.978), exceeding the critical value of 3.84 (*df* = 1), indicating that the individualism-moderated model provided a better fit. The interaction term significantly predicted negative affect (*β* = 0.12, 95% CI = [0.11, 0.13], *p* < 0.001). However, its effect on positive affect was not significant (*β* = 0.01, 95% CI = [−0.01, 0.02], *p* = 0.524). Similarly, the model with the interaction between compliance and collectivism (see Figure 4b) also produced a significant log-likelihood ratio (*D* = 36.13), exceeding the 3.84 threshold (*df* = 1), suggesting that the collectivism-moderated model was superior to Model 0. However, the interaction term between compliance and collectivism did not significantly moderate the effect on either positive affect (*β* = −0.01, 95% CI = [−0.03, 0.01], *p* = 0.308) or negative affect (*β* = 0.01, 95% CI = [0.00, 0.02], *p* = 0.110).

Simple slope analysis revealed that the positive association between compliance and negative affect was stronger for high individualism (+1 SD; *b* = 0.71, 95% CI = [0.68, 0.73], *p* < 0.001) than for low individualism (−1 SD; *b* = 0.45, 95% CI = [0.42, 0.47], *p* < 0.001) (see Figure 5b). This indicates that individualism significantly moderated the relationship between compliance and negative affect. Furthermore, the conditional indirect effect of compliance on psychological distress via negative affect was also stronger for high individualism (*b* = 0.24, 95% CI = [0.23, 0.26], *p* < 0.001) than for low individualism (*b* = 0.15, 95% CI = [0.14, 0.16], *p* < 0.001).

### 3.6. Sensitivity Analyses

To assess the robustness of our findings, we conducted two sets of sensitivity analyses. First, we re-estimated all primary models with additional controls for psychiatric history and medication use. The results remained virtually unchanged: all core effects remained significant, and effect sizes differed by no more than 0.01 from those in the main models (see Table S1 in the Supplementary Materials for detailed comparisons). Second, we re-estimated all core models using a latent affective distress factor, comprising the depression and anxiety subscales of the SCL-90, as the outcome variable in place of the global severity index. The results remained largely consistent with those based on the SCL-90 Global Severity Index (GSI) (see Table S2 for detailed comparisons). Taken together, these sensitivity analyses confirm that our findings are robust.

## 4. Discussion

The findings reveal a significant positive association between compliance and psychological distress. This suggests that individuals with higher levels of compliance may experience greater psychological distress in China, aligning with previous findings from Western research (Gudjonsson & Main, 2008; Gudjonsson et al., 2002). From a self-determination theory perspective, this cross-cultural consistency may reflect a relatively universal mechanism: habitually prioritizing others’ demands over one’s own authentic preferences frustrates the basic need for autonomy (Ryan & Deci, 2017), which is in turn associated with greater vulnerability to psychological distress (Ryan & Deci, 2000).

The results also indicate that affects play a significant mediating role in the association between compliance and psychological distress. Specifically, both positive and negative affect mediated this association, consistent with the view that positive and negative affect are orthogonal rather than opposite (Crawford & Henry, 2004; Watson & Tellegen, 1985). Notably, the mediating role of negative affects was stronger than that of positive affect, suggesting that the negative affect may play a more prominent mediating role in the compliance–distress link.

Regarding cultural values, the findings diverge from previous research. While past studies suggested that individuals in collectivist cultures tend to exhibit higher levels of compliance (Bond & Smith, 1996; Oh, 2013), this study found that the correlation between compliance and individualism or collectivism was not significant. This discrepancy may be explained by two factors: First, all participants in this study were recruited from China, a predominantly collectivist society. Thus, individual-level cultural values measured within a predominantly collectivist context may primarily reflect individual variations in cultural values rather than the overall societal culture. In other words, within China’s overarching collectivist cultural framework, the influence of individual-level individualism or collectivism on compliance may be minimal. Second, during the process of socio-cultural transformation, collectivist and individualistic orientations often coexist (Triandis, 1996), which may complicate the relationship between compliance and cultural values. Interestingly, the study results indicate that individualism does not directly predict compliance but does moderate the effects of compliance on psychological distress and negative affect. This finding has rarely been reported in the previous literature and may provide new insight into the role of cultural orientation in the psychological consequences of compliance.

This study found that, within a Chinese collectivist cultural context, individual-level individualism and collectivism independently moderated the relationship between compliance and psychological distress. Specifically, the positive association between compliance and psychological distress was stronger among individuals with higher levels of individualism, whereas collectivism did not moderate this relationship. Similarly, individualism also moderated the mediating role of negative affect. Specifically, the indirect effect via negative affect was stronger for those with higher levels of individualistic values. Collectivism, however, did not significantly moderate either the direct or indirect effect. One possible explanation is that, for young adults in a collectivist-oriented society, collectivistic values may be adopted out of necessity rather than personal choice, as the broader social environment emphasizes conformity to social norms (Cialdini et al., 1999). In such contexts, individuals’ endorsement of collectivism may be shaped more by external societal expectations than by intrinsic personal desires. Consequently, these externally driven values may neither serve as a positive psychological resource nor effectively buffer the relationship between compliance and negative affect or psychological distress.

### 4.1. Implications

This study makes interdisciplinary contributions by integrating perspectives from personality, socio-cultural, and health psychology, thereby providing a foundation for future research on compliance. First, by distinguishing situational compliance from trait compliance, this study strengthens the theoretical framework for understanding compliance as a stable disposition. Second, this study elucidates the roles of positive and negative affect in the association between compliance and psychological distress. Third, it identifies individual-level cultural orientations as moderators of the association between compliance and psychological distress within the Chinese context. Specifically, the association between compliance and psychological distress was more pronounced among individuals with higher levels of individualism.

In addition, the observed effect sizes carry practical implications. The medium-sized correlation (*r* = 0.40, 16% variance) indicates that trait compliance is a meaningful risk factor, encouraging practitioners to consider it in assessment and intervention. The indirect effect via negative affect (*β* = 0.23), accounting for 43% of the total effect, underscores the clinical relevance of affective pathways. This finding suggests that interventions targeting affective regulation may help mitigate the mental health costs of excessive compliance. Furthermore, the findings highlight the importance of considering individual differences in cultural values when designing clinical interventions, particularly for individuals with stronger individualistic orientations, as the compliance–distress link is more pronounced in this group.

### 4.2. Limitations and Future Directions

Several limitations of this study warrant consideration. First, the exclusive reliance on self-report measures raises concerns about common method bias, which may have inflated the observed relationships among variables. Future studies could enhance validity by incorporating multi-method approaches, such as behavioral observations, peer or family informant reports (especially when assessing dispositional compliance), or physiological indicators (e.g., sleep patterns, physical activity) that provide objective correlates of psychological distress. Second, the cross-sectional design precludes causal inference. Future research could adopt multi-wave longitudinal designs using cross-lagged panel models to clarify causal directions, or experimental manipulations of compliance (e.g., social pressure) to assess immediate changes in affect and well-being. Third, we did not control for personality dimensions such as neuroticism, agreeableness, or self-esteem, which may have confounded the compliance–distress association. Future research should include comprehensive personality measures as covariates to isolate the unique contribution of compliance. Finally, this study examined the individual-level cultural orientations rather than cross-cultural differences. Future research could address this gap by including participants from diverse cultural backgrounds (e.g., China and the United States) to test whether the observed patterns differ across societies.

## 5. Conclusions

This study offers a novel perspective on compliance by focusing on its dispositional nature and its implications for psychological distress. Our results indicated that compliance was associated with increased negative affect and decreased positive affect, which are in turn associated with higher levels of psychological distress. Crucially, we observed that the shared association between compliance and psychological distress, particularly through negative affect, was distress among individuals with higher levels of individualism. This study enriches our understanding of the interplay between compliance and psychological distress, advances theoretical knowledge in health psychology, and provides valuable insights for the development of culturally tailored clinical interventions for psychological distress.

## Figures and Tables

**Table 1:** Demographic characteristics of the sample (*N* = 11,038).

Variables	Mean ± SD/n (%)
Age (years)	20.58 ± 4.11
Sex	
male	3683 (33.4%)
female	7355 (66.6%)
Ethnic	
Han	9692 (87.8%)
Other	1346 (12.2%)
Educational level	
High school and below	143 (1.3%)
Undergraduate	6632 (60.1%)
Masters	4024 (36.4%)
PhD	239 (2.2%)
Type of residence	
City	4722 (42.8%)
Town	5289 (47.9%)
Village	1027 (9.3%)
Whether an only child	
Yes	6340 (57.4%)
No	4698 (42.6%)
Psychiatric history	
Yes	308 (2.8%)
No	10,730 (97.2%)
Current medication intake	
Yes	45 (0.4%)
No	10,993 (99.6%)
Family economic level	2.95 ± 0.52
Subjective social class	4.78 ± 1.56
Compliance	50.83 ± 11.12

## Data Availability

The datasets used and analyzed during the current study are available from the corresponding author on reasonable request.

## Supplementary Materials

The following supporting information can be downloaded at: https://www.mdpi.com/article/10.3390/bs16071187/s1, Table S1. Comparison of effect sizes between the main model and the sensitivity model with additional covariates (psychiatric history and medication use). Table S2. Comparison of core path coefficients between the main model (SCL-90 GSI) and the latent affective distress model (depression and anxiety subscales).
