# Supplementary material for: When Saying “Yes” Hurts: The Relationship Between Compliance and Psychological Distress Across Cultural Values in China
Source: Behav Sci (Basel). 2026 Jul 14;16(7):1187. doi: 10.3390/bs16071187 (PMC13405851; doi:10.3390/bs16071187)
Supplement: Supplementary file 1 [file behavsci-16-01187-s001.zip › behavsci-4365171-supplementary.pdf]

Supplementary Materials

Social-demographic questionnaires

Participants' sociodemographic information was collected using a self-developed questionnaire comprising several items. The variables assessed included age, sex, ethnicity, educational level, type of residence, only-child status, psychiatric history, current medication intake and family economic level. Age was measured using an open-ended item allowing participants to report their age in years. Sex was assessed using a binary categorical item ("What is your sex? 1 = Male, 2 = Female"). Ethnicity was captured through a dichotomous classification ("Your ethnic: 1. Han, 2. Other"). Educational level was evaluated with an ordinal scale ("Your educational level: 1. Preparatory, 2. Undergraduate, 3. Masters, 4. PhD"). Type of residence was assessed through a nominal item with three response options ("What is your type of residence? 1 = Urban, 2 = Town, 3 = Rural"). Only-child status was determined through a binary item ("Are you an only child? 1. Yes, 2. No"). Psychiatric history was assessed using a yes/no item ("Have you ever been diagnosed with a psychiatric disorder? 1 = Yes, 2 = No"), and current medication use was measured similarly ("Are you currently taking any psychiatric or related medications? 1 = Yes, 2 = No"). Family economic level was assessed via a 5-point Likert-type scale ("Your family economic Level: 1 = Very wealthy, 2 = Wealthy, 3 = Average, 4 = Poor, 5 = Very poor").

3.6 Sensitivity Analysis

Table S1. Comparison of effect sizes between the main model and the sensitivity model with additional covariates (psychiatric history and medication use).

Path	Main model β	Sensitivity model β (with additional covariates)
Compliance → Psychological distress	0.42***	0.41***
Compliance → Negative affect→ Psychological distress	0.23***	0.23***
Compliance → Positive affect→ Psychological distress	0.05***	0.05***
Compliance × Individualism→ Psychological distress	0.06***	0.06***
Compliance × Individualism→ Negative affect	0.12***	0.13***

Note: All models adjusted for age, sex, ethnicity, education level, and subjective social class. Sensitivity models additionally controlled for psychiatric history and medication use. *** $p < .001$

Table S2. Comparison of core path coefficients between the main model (SCL-90 GSI) and the latent affective distress model (depression and anxiety subscales).

Path	Main model β (SCL-90)	Sensitivity model β (Depression and anxiety)
Compliance → Psychological distress	0.42***	0.43***
Compliance → Negative affect→ Psychological distress	0.23***	0.25***
Compliance → Positive affect→ Psychological distress	0.05***	0.07***
Compliance × Individualism→ Psychological distress	0.06***	0.10***
Compliance × Individualism→ Negative affect	0.12***	0.12***

Note: All models adjusted for age, sex, ethnicity, education level, and subjective social class
*** $p < .001$
